# Resurrecting the Dead (Molecules)

**DOI:** 10.1016/j.csbj.2017.05.002

**Published:** 2017-05-30

**Authors:** Jan Zaucha, Jonathan G. Heddle

**Affiliations:** a Departament of Computer Science, University of Bristol, Life Sciences Building, 24 Tyndall Avenue, Bristol BS8 1TQ, United Kingdom; b Bionanoscience and Biochemistry Laboratory, Jagiellonian University, Malopolska Centre of Biotechnology, Gronstajowa 7A, 30-387 Kraków, Poland

## Abstract

Biological molecules, like organisms themselves, are subject to genetic drift and may even become “extinct”. Molecules that are no longer extant in living systems are of high interest for several reasons including insight into how existing life forms evolved and the possibility that they may have new and useful properties no longer available in currently functioning molecules. Predicting the sequence/structure of such molecules and synthesizing them so that their properties can be tested is the basis of “molecular resurrection” and may lead not only to a deeper understanding of evolution, but also to the production of artificial proteins with novel properties and even to insight into how life itself began.

## 1 Introduction

The idea that species may no longer exist, having become extinct through catastrophic events, competition or simply evolution into new species is a familiar one and also applies to biological molecules. Trivially this is true – the genomes of extinct species, for example, clearly no longer functionally exist. It is not necessarily true of *all* molecules from extinct species of course: Some may continue to function as identical or near-identical versions in related organisms. In contrast, there may be unique biological molecules that no longer form part of any living system (some DNA/RNA sequences and proteins being of particular interest) and their study could uncover new information regarding evolutionary pathways as well as allowing us to discover novel molecules with useful functions.

There are at least two approaches to resurrecting extinct biological molecules: one is through their extraction from the environment, i.e. the discovery of molecular fossils. Recovering ancient biological molecules in this way (so-called “molecular palaeontology”) relies on them being amenable to long-term preservation. Clearly, less stable molecules (for example RNA in contrast to DNA) are less likely to be preserved over long periods with the exact time depending on environmental conditions: DNA itself has been estimated to have a half-life of 500 years in bone for a 30 bp fragment at 25 **°**C [1]. There are of course rare exceptions where conditions such as consistent low temperatures can preserve samples for longer periods, for instance, in permafrost. This has allowed complete mitochondrial DNA over 100 k years old to be recovered from a polar bear jawbone [2]. Even more impressively, ancient horse DNA from a bone over 500 k years old preserved in permafrost has been recovered and the genome sequenced, resulting in a wealth of insight into the evolution of modern horses [3]. The oldest authenticated DNA, which has been extracted from the basal sections of deep ice cores in Greenland, has been dated to be 450-800 k years of age [4]. This may be close to the temporal limit of DNA recovery from fossils. Although traces of the molecule have been detected in dinosaur specimens millions of years old [5], it is unlikely that the samples can yield information-bearing sequences [6].

In comparison to DNA, polypeptides within fossils have an even lower degradation rate, which allows for their recovery from more ancient samples and when shielded from weathering, have been preserved for millions of years.

Finally, the most persistent biomolecules able to provide meaningful insights about the inhabitants of the ancient world, are simple biopolymers such as the pigment melanin [6]. It has been demonstrated to be capable of surviving in fossils originating even from the early Jurassic era (older than 175 m years) [7].

The second approach to resurrection of biological molecules is to use *in silico* methods to *predict* their identity and then produce them synthetically. In the case of genomes, this could allow “genome transplantation” to recover a functioning organism. Indeed a bacterial genome has been completely synthesized and used to “reboot” a cell [8]. Such a method may of course be open to inaccuracies but attempts to resurrect ancient proteins in this way have led to interesting results including the production of novel molecular structures, which may prove to be useful tools in unexpected areas such as bionanoscience.

In this work, we will review both molecular palaeontology and bioinformatics approaches to determining the identities of extinct DNA and protein molecules and the proven and potential usefulness of such information including as a biotechnological tool.

## 2 Molecular Palaeontology

### 2.1 Rescuing DNA Fossils

The relatively high stability of DNA means that under favourable conditions it can be preserved for extended periods of time [1]. Recovered DNA sequences allow insights into evolution, giving an understanding of how an extinct species fits into the tree of life. If whole genomes are recovered it raises the prospect of “de-extinction” [9] and, using recombinant DNA technology allows production, purification and characterization of proteins encoded within the DNA (see Section 3).

Recovering DNA from ancient samples of extinct species is a difficult task due to potentially limited amounts of sample, problems of contamination and the deterioration of the molecules over time. In addition, post-mortem DNA is subject to deterioration including fossil weathering and degradation by microorganisms resulting in DNA fragmentation, while oxidative lesions can affect both the nucleotide bases and the deoxyribose sugar residues. If unaccounted for, these may result in spurious results and render the ancient DNA difficult to sequence due not least to contamination by present-day, damage-free samples that yield stronger signals [10].

The first obstacle i.e. amount of sample, has been mitigated by the development of the famous polymerase chain reaction [11], which allows for the amplification of molecular content even from the smallest amounts of specimen [12], [13]. Next-generation sequencing techniques can then provide high quality full coverage genome sequencing [14]. Problems of physical damage can be overcome by independent deep sequencing of short overlapping fragments from multiple sample clones [15].

Another challenge is the formation of inter-strand and intermolecular DNA crosslinks through interactions between DNA strands or DNA and other biomolecules (e.g. proteins). These are the products of alkylation or Maillard reactions respectively and can be mitigated with cross-link breakers such as *N*-Phenacylthiazolium bromide [15], [16]. However, hydrolytic lesions (also referred to as type II damage) are the most important to account for, since they alter the genetic code in the specimen with respect to the host's original DNA. The most commonly observed artefact results from the deamination of cytosine to uracil, which is chemically analogous to thymine. Depending on the DNA strand (forward or reverse), this causes an apparent G/C to A/T single nucleotide polymorphism [17]. Further lesions include the substitution of adenine to hypoxanthine, 5-methyl-cytosine to thymine and guanine to xanthine [15]. In second-generation sequencing technologies, such as the Illumina and Solexa platforms, whole ensembles of DNA molecules are ‘washed-and-scanned’ for using previously generated libraries of molecules [18]. In order to achieve full coverage of the ancient genome – and reduce the risk of contamination – the sequencing libraries need to be prepared in line with the expected chemical distribution of the ancient DNA [19]. Otherwise, the sequencing will result in a lower (and biased) read yield [20]. Alternatively, the use of the so-called third-generation single-molecule sequencing technologies [18], which do not rely on library scanning, allows for a straightforward ancient genetic data extraction. However, in contrast to second-generation sequencing, the precious sample cannot be re-amplified, thus a combination of second-generation and singe-molecule sequencing has been suggested as the best solution [20]. The final noteworthy point is that the occurrence of such DNA degradation patterns is to be expected of ancient specimens and can be used as evidence for genuine paleo-genomes (in contrast to contaminants) [21], [22]. With this in mind, ancient genetic data obtained with Next Generation Sequencing techniques can be analysed using the mapDamage2.0 software package, which includes a Bayesian statistical framework accounting for the basic expected types of post-mortem DNA damage [23].

Despite the above-mentioned difficulties, significant progress in recovering and sequencing ancient DNA from extinct species has been made; as early as 1984 small amounts of DNA from an extinct subspecies of zebra (the quagga) were recovered from the dried muscle of a museum specimen [24]. More recently, the genome sequence of the woolly mammoth (*Mammuthus primigenius*) has been reported [25] leading to the recreation of the animal's haemoglobin using a recombinant approach [26]. Amino acid substitutions in the ancient protein have been found to confer an adaptation for the harsh Pleistocene environment providing a higher efficiency of oxygenation in lower temperatures, as compared with the extant transcripts found in the mammoth's currently living closest relative – the Asian elephant. A crystal structure obtained for the protein elucidated the mechanism of its altered characteristics revealing small structural changes significantly affecting the affinity for oxygen [27].

Closer to home, recovering DNA from ancient members of the human lineage has enriched understanding of our own family tree and patterns of migration [28]. DNA (mitochondrial) from Neanderthals was first recovered and sequenced in 1987 [29]. Further DNA sequences from Neanderthal remains were subsequently recovered [14], [30], [31], [32] leading recently to the full genome sequence reported for an over 50,000 years old toe bone from a female Neanderthal woman found in Denisova cave, Siberia [33]. The same cave has also allowed recovery of DNA from and identification of a new relative of modern humans, the so-called “Denisovans” [22], [34]. It is now known that Denisovan DNA contributed significantly (up to 5%) of the DNA of current Oceanic peoples [35].

The availability of full genome sequences of extinct species raises the prospect that some could be subject to “de-extinction” [9] an idea that has most often been discussed in relation to large animals (typically mammals). Most simply this would require not only the relevant DNA sequences but also intact nuclei to allow somatic cell nuclear transfer (SCNT) [36] into the egg cell of a close living relative. SCNT is now a relatively common technique in cloning and has been carried out using nuclear material from extinct animals including an attempt to resurrect the Pyrenean ibex [37]. This was partially successful: the resulting offspring was born alive but only survived for a few minutes. For more ancient extinct species, where an intact nucleus may not be recoverable, the process will be more challenging: Without whole nuclei, SCNT is not feasible and genomes may have to be produced purely synthetically and introduced into recipient cells. The first challenge – production of synthetic genomes is developing rapidly [38]: synthesis of a whole eukaryotic chromosome has been reported [39] and work is underway to produce a whole synthetic yeast genome [38] with the final design recently reported [40]. The second challenge, provision of a suitable recipient cell, seems a more distant prospect as such cells may not exist or if they do, their use may raise ethical questions (such as when the donors of the cells are themselves endangered) [41]. Overall, using genomic DNA from extinct animals for the purposes of de-extinction still has significant scientific challenges before it can be considered generally useful.

### 2.2 Rescuing Protein Fossils

Ancient protein has been recovered from extinct species on several occasions. This may be less attractive than recovering DNA sequences as DNA can be amplified, sequenced and then used to produce large quantities of protein via a recombinant approach. Nevertheless in very old samples, recovery of full genomes is not expected and so direct recovery of proteins, which exhibit lower degradation rates than DNA, has merit [42]. Advancements in the field of mass-spectrometry, tailored towards the recovery of protein sequence from even the smallest amounts of ancient specimens have opened new opportunities [43]. Knowledge of protein sequence can, for example, be used to infer evolutionary relatedness between organisms and using protein sidesteps contamination issues that may be problematic when recovered DNA samples are amplified. The virtues and challenges of ancient protein recovery are numerous [44], but evidence of the potential of proteins to be preserved for extreme lengths of time has been shown in work where peptide sequences were extracted from ostrich shells dated to be 3.8 m years old [45], and sequences of collagen have been recovered from 3.4 m years old camel bones, allowing comparison with other ancient and existing species [46]. Even partial sequences of bone matrix and vessel proteins from 60 to 80 m years old dinosaur specimens have been reported [5], [47], [48], [49], [50] and while, the validity of the results has been questioned [45], recently, some of them have been reproduced using an independent experimental procedure [51].

The recovery of proteins is not limited to the extraction of single peptides, but allows entire proteomes to be obtained, providing good evidence that DNA sequences were actually transcribed and translated into functional proteins that built an ancient organism, such as the obtained proteome from the woolly mammoth's femur [52]. More importantly however, proteomes can provide richer information than DNA sequence alone. In particular, they offer a tissue-specific snapshot of the transcriptome [42] i.e. gene expression levels at the time of death, providing insight into the circumstances of the host's death such as the presence of a severe bacterial infection indicated by the immune response detected in the proteome of a 500 years old Incan mummy [53].

## 3 Bioinformatics Approaches to DNA and Protein Resurrection

Discovering the sequence of ancient genes does not necessarily require rescuing actual ancient molecules of DNA: Bioinformatics techniques can be used to calculate the likely sequence of ancient genes based on existing sequences. Knowledge of the types of genes present in ancient organisms can give information not just about the organism itself but the kind of environment it lived in. Such an approach is necessary in most cases as access to ancient specimens is often limited. Doing so relies on knowledge of the molecular content of extant species (fully sequenced genomes) and their classification in order to reconstruct phylogenetic relationships in the form of an evolutionary tree.

Phylogenetic tree reconstruction has been the topic of a number of comprehensive reviews in its own right [54], [55], [56], [57]. The basic principles are that the input to any algorithm is sequence data for each species. In order not to introduce biases, only completely sequenced genomes should be considered. Despite the often-dramatic sequence divergence, protein structure is conserved and distant homology may be detected using profile Hidden Markov Models, which outperform other methods [58], [59]. Therefore, for the purpose of phylogenetic reconstruction, amino acid sequences are preferentially used over gene sequences [60]. It is important to note that various species have been sequenced to different levels of quality due to the application of different sequencing technologies, read depths and data analysis techniques. Only high quality datasets should be included; the Proteome Quality Index provides a means of filtering and downloading proteomes from all complete sequencing projects [61].

Furthermore, within proteins, conserved structural units, referred to as domains, have been identified. The Structural Classification of Proteins (SCOP) provides a hierarchical classification of protein domains into families and superfamilies [62]. The family classification approximates traditional sequence-only phylogenetic reconstruction techniques [63], while domains within a superfamily are thought to share evolutionary descent and are regarded as basic units of evolution [64]. Most full-length proteins are built up of a linear combination of structural domains - referred to as domain architectures – that are capable of performing highly specialized functions in concert and each domain can be a building block of a variety of different proteins [65]. For the purpose of phylogenetic reconstructions, the most important feature of domains (as well as domain architectures) is that they are less likely to be the result of homoplasy than their counterpart full-length protein transcripts [66]. Therefore, they are considered more reliable in detecting distant homology and have been advocated for use as input into tree phylogenetic building algorithms [67]. Protein domain annotations for any amino acid sequence can be obtained from the SUPERFAMILY database for SCOP domain definitions [68] and alternative domain definitions are provided by the CATH database [69].

Annotated proteomes from complete sequencing projects provide binary ‘presence’ or ‘absence’ flags for clusters of homologous molecular features, such as protein domain families and superfamilies, domain architectures or orthologous transcripts, for use as the input for the phylogenetic tree inference algorithm of choice. The most widely used algorithm is Randomized Accelerated Maximum Likelihood (RAxML), which applies the optimality criterion of maximum likelihood, but features speed optimizations (most importantly, the initialization of several starting trees to avoid being trapped in local maxima) and computational parallelization in order to allow computing the most likely tree topology (a problem classified as NP-hard) even for large datasets [70].

A daily-updated (after the addition of new proteomes from complete sequencing projects) sequenced Tree of Life (sTOL) [67] has been implemented using the procedure outlined above with the addition of a likelihood weight calibration algorithm that consolidates the SUPERFAMILY annotated molecular content of all completely sequenced organisms [68] with their respective NCBI taxonomic information [71].

A similar approach, although relying on full-length proteins (grouped by Markov Chain Clustering [72] of reciprocal BLAST hits [73]), rather than domain annotations, has been applied recently to infer the molecular content of LUCA (the Last Universal Common Ancestor of extant organisms), which resides at the very root of the phylogenetic tree, wherefrom bacteria and archaea descend (recent research suggests the existence of only two primary domains of life [74]). Under the selected assumptions (amino acid sequence cluster monophyly and the presence of a cluster member in at least two representatives of both bacteria and archaea) 355 protein sequence clusters have been identified as key molecules stemming from LUCA. This contrasts with a demonstrated minimal bacterial genome of 473 genes [75], highlighting the fact that computational inferences are capable of robustly identifying only the most highly conserved features of the ancestor state. The established conserved proteins of LUCA place it closest to the extant clostridia and methanogens amongst bacteria and archaea respectively and their distribution gives hints about LUCA's physiology and habitat [76].

**Fig. 1:**
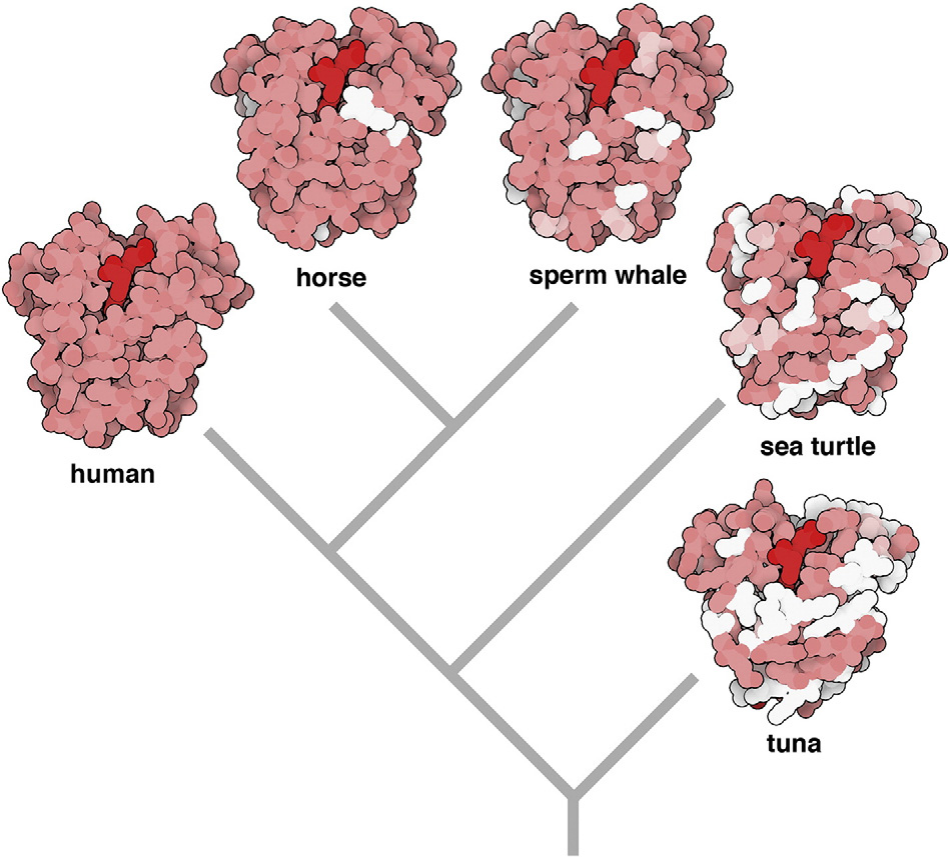
Phylogenetic reconstruction based on myoglobin sequences: evolutionary relatedness between species was reconstructed using the Phylogenics.fr web server [87]. The colours show differences to the human transcript, which is used as reference; pink residues indicate identical amino acids; residues similar in physio-chemical properties are light pink; vastly different side-chains are white. The heme is shown in bright red. PDB structures depicted: human (3rgk [115]), horse (1ymb [116]), sperm whale (1mbo [117]), sea turtle (1lhs [118]), tuna (2nrl [119]). This image was originally generated by David S. Goodsell for the RSCB PDB Molecule of the Month, February 2017 [88] and is available at the RCSB PDB [120]. (For interpretation of the references to colour in this figure legend, the reader is referred to the web version of this article.)

Inferring a phylogenetic tree based on extant proteomes allows establishment of the most likely evolutionary relatedness between species and the set of conserved proteins originating from each ancestral node, without determining their true identity in the past. Sequence information is generally available only for extant transcripts, located at the leaves of the phylogenetic tree. Despite an overall conservation, all sequences diverge from their ancestor states due to evolutionary drift. With the rare exceptions of species where specimens have been preserved, allowing for the recovery of their ancient biomolecules – such as the woolly mammoth's haemoglobin [27], resurrected from its respective gene [26] (see above) - ancient polypeptide sequences need to be inferred at each ancestral node [77]. Although this can be straightforwardly obtained from the consensus sequence of extant domain representatives descending from the clade node [78], such inference is highly sensitive to the number of species accounted for in the calculation. More accurate methods rely on the topology of the associated phylogenetic tree, which can be inferred from the multiple sequence alignment of extant versions of the protein sequence by themselves, or provided as input for the algorithm if the tree has already been determined otherwise, for example by considering the full proteome of the hosts. The maximum-parsimony approach assigns the residues at the ancestral nodes so as to minimize the number of amino acid substitutions. It allows correct determination of the true ancestral state provided that there is sufficient sequence similarity between proteins located at the leaf nodes. However, in the case of high sequence divergence, the maximum likelihood approach has been repeatedly shown to be the most reliable method [79], [80]. Taking into account tree branch lengths as well as an evolutionary model characterizing mutagenesis, it yields the most likely ancestral sequence unambiguously. Furthermore, the Bayesian framework can be invoked to calculate the probability of each possible ancestral state providing confidence values for the results [81]. The task is now easily achievable for non-experts, as largely automated platforms for protein phylogenetic inference have been implemented [82], [83], [84], [85], [86]. Such reconstructed relatedness of myoglobin sequences is presented in Fig. 1 [87], [88].

## 4 Hybrid Approaches

Hybrid approaches fuse together bioinformatics and molecular biology to predict ancient protein sequences and then produce them: Amino acid residues can be reverse translated into nucleotide codon triplets (subject to codon optimization for the organism used for expression) to arrive at the gene sequence, which can be synthesized and cloned into a plasmid for high level expression [60]. Such an approach has been applied to resurrect ancient bacterial elongation Tu-factors and establish from their properties the palaeo-environment from over a billion years ago [89] as well as to analyse the evolution of currently functionally distinct steroid hormone receptor proteins, determining their ancestor as the estrogen receptor [90]. It has even been applied to dinosaur rhodopsin visual pigment suggesting adaptation to low light levels [91].

Furthermore, in addition to sequence analysis, conserved structural and functional features, i.e. protein domain superfamilies [62] - taken as the unit of evolution, as well as short linear motifs (SLiMs) that act as links in molecular pathways [92], can also provide insight into the identity of ancient molecules. In the light of evolution, structural homology of functional molecules across species is unsurprising. However, counter-intuitively, related structures often have a very low sequence identity, for example the broad family of globins has an average sequence identity of 17% [93], despite that, their tertiary structures differ only in minor details (Fig. 1). The core of the protein determines how the protein folds and usually serves the role of the functional active site. It is the part of the sequence that remains best conserved, often forming an easily identifiable motif (or a linear combination thereof). On the other hand, the particular side chains of the remaining sequence residues across members of a superfamily are usually less specific; the conservation of their physio-chemical properties, such as electric charge or hydrophobicity is generally sufficient to preserve the domain's structure [94]. The creation of new domains is rare. Biological complexity is driven by domain duplication and rearrangement (through gene duplication and the emergence of new splice variants) as well as the specialization of particular units through selecting for motifs formed after the accumulation of benign mutations [95]. SLiMs on their own, although less extensively studied than domains and less reliable as evidence for functionality [96], can be nevertheless identified within a transcript (especially within eukaryotes [97]) and serve the role of a definition for a functional feature traceable through evolution. A phylogenetic reconstruction of features within a protein, which over the course of time has altered its domain content or accumulated new functional motifs, can allow tracing evolution backwards and recovering the key structural and functional characteristics of the ancestral molecule, despite not knowing its exact amino acid sequence.

**Fig. 2:**
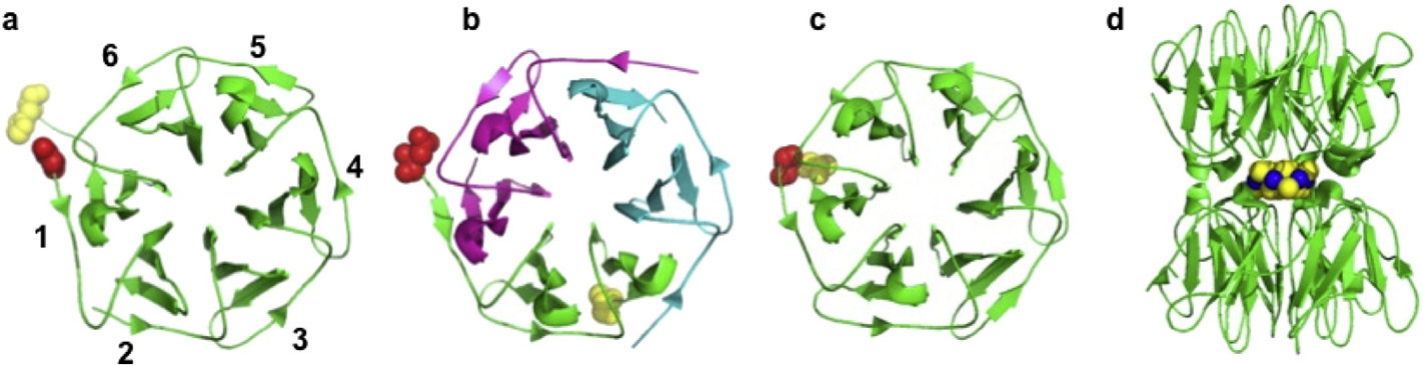
Resurrected proteins in protein engineering: a), b) and c) show the crystal structures of an extant β-propeller protein (1rwl [99]) and two engineered versions (3ww7 and 3ww9 [98]) respectively. In each case the structures are shown in cartoon format with each continuous polypeptide chain in a single colour. The N- and C-termini of one peptide chain in each structure are shown in red and yellow sphere depiction respectively. In a), each of the six blades is numbered, b) and c) are constructed of an identical “ancestral” blade with c) being formed from 3 copies of a single polypeptide “dimer” of blades and c) being constructed from a single polypeptide consisting of 6 copies of the blade and d) shows a nanocrystal of CdCl_2_ shown as spheres (blue = Cd, yellow = Cl) between two copies of a designed Pizza2 protein (nvPizza2-S16H58, pdb 5chb) [107]. The Pizza protein rings are shown in an orthogonal view and at a smaller scale than a-c. (For interpretation of the references to colour in this figure legend, the reader is referred to the web version of this article.)

An intriguing example of a hybrid approach comes from the work of Voet et al. [98], summarised in Fig. 2. They took a domain of protein kinase from *Mycobacterium tuberculosis* (NHL repeat structure, PDB entry 1RWL [99]) which forms a β-propeller domain made from six highly similar but not identical “blades”. The number of blades in a β-propeller protein varies depending on the protein but all blades are similar, consisting of a short sequence forming a β-sheet [100], [101] with 1RWL consisting of six such blades. The similarity of each blade in any given propeller protein suggests that they evolved by gene duplication and fusion from an original gene corresponding to a single blade. Voet et al. used a bioinformatics approach to approximate several putative sequences of this “ancestor” blade protein [102]. These sequences were next evaluated utilizing a Rosetta [103] based computational protein design algorithm to identify the sequence most compatible with a perfect symmetrical β-propeller architecture. This resurrected protein shows that six identical repeats fused into a single protein can fold into the 6-bladed propeller. This protein (referred to as Pizza due to its appearance) proved to be highly thermostable and it is interesting to note that the apparent trend for ancient proteins to exhibit increased stability has been recently reviewed [104]. Variations with different number (2–10) of repeats were observed to self-assemble into larger complexes with a total number of repeats equalling the lowest common multiple of 6 and the number of tandem repeats, showing the high tendency to assemble into the 6-bladed architecture.

The work demonstrated a mix of techniques [102]: Bioinformatics was used to search structural databases for all known six-bladed β-propeller proteins, which were manually assessed to find an attractive candidate. The sequences of the six blades of 1RWL were submitted to the FastML webserver for ancestral protein sequence prediction [83], which suggested that blade three was the closest match to the original ancestral protein. This blade was used to model a perfectly 6-fold symmetrical propeller protein using RosettaDock [105] and potential ancestor sequences were modelled onto this scaffold using a PyRosetta [106] based procedure and the lowest energy structures were identified [102]. Molecular biology techniques were used to synthesize the gene encoding Pizza protein and the recombinant protein was produced and purified and the crystal structure determined, which confirmed that its structure matched that predicted. The high stability and symmetrical nature of the protein have made it amenable to further engineering: It was subsequently modified into a variant able to biomineralise nanocrystals of cadmium chloride [107]. It has even been speculated that Pizza protein may be a suitable platform for design of synthetic enzymes [107].

## 5 Summary and Outlook

Advances in technology have allowed us to locate and recover proteins and DNA from ancient specimens and determine their sequences. Genomic data has given us a glimpse of extinct species, ancient environments and the tree of life. Ultimately there are limits to the quantity, quality and age of molecules that we can expect to recover and here computational approaches will be important. Already, bioinformatics has enabled prediction of a possible proteome of LUCA, as described above. This points to a complex entity comprising all three basic types of bio-molecules (DNA, RNA and proteins) as well as lipid membranes providing the molecular machinery required for an efficient metabolism and cellular compartmentalization [76], [108]. Given the spontaneous emergence of life, primordial molecules must have been much simpler. For example, short polypeptide sequences have been demonstrated to have functional capabilities relevant to the prebiotic world [109].

**Fig. 3:**
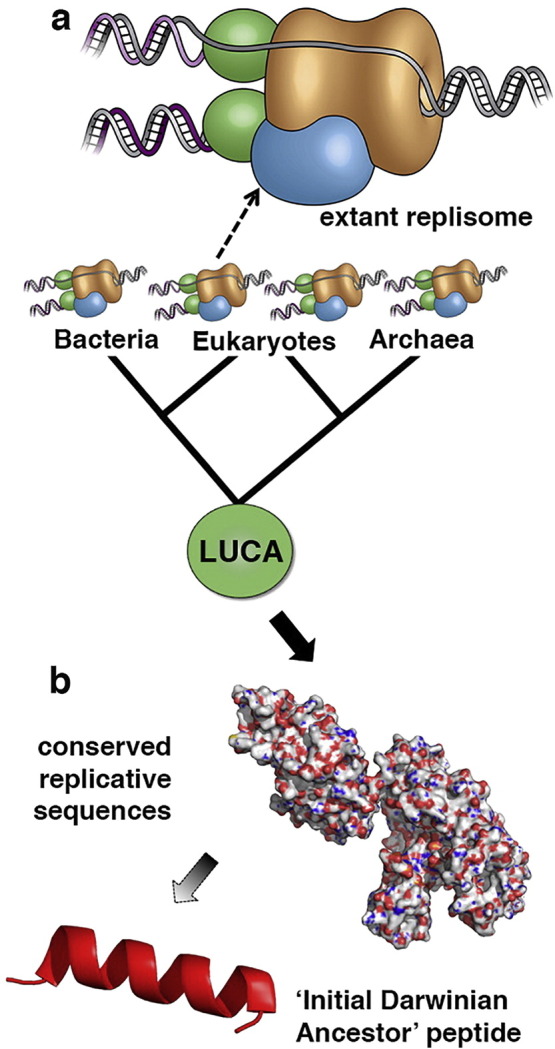
Resurrection of the Initial Darwinian Ancestor: a) representatives of extant replisome complexes [121] at the leaves of the phylogenetic tree allow tracing evolution backwards to identify the key replicative molecules conserved from the age of LUCA and infer their maximum likelihood sequences. b) The replicative proteins of LUCA may harbour within their sequences, motifs stemming from the primordial “Initial Darwinian Ancestor”, the self-replicating precursor to life. Here the “conserved replicative sequence” is represented for illustrative purposes only, by a DNA polymerase (1tau [122]); the “Initial Darwinian Ancestor” peptide is represented by a helical sequence extracted from a larger protein structure (2ZP8 [123]) and is used for illustrative purposes only. Replisome cartoon shown in a) is courtesy of the Brookhaven National Laboratory.

Is it possible to trace evolution even further back and “discover” the molecules that predate LUCA and cellular life itself? Despite the colossal molecular innovation between LUCA and the very first self-replicating system – the “Initial Darwinian Ancestor” (IDA) [110], its crucial replicative functionality must have been preserved to ensure a continuous lineage. It may be that current replisomes still harbour remnants of the primordial IDA, which can be identified by selecting for parts of sequences that satisfy maximum parsimony, working down to the root of the phylogenetic tree (Fig. 3). The discovery of such a replicator system through analysis of extant sequences would be of great scientific interest as the presence of the molecule within living species would be direct evidence of its identity as the precursor to life.

At the other end of the complexity scale, a knowledge of whole genome sequences from extinct species inevitably leads to the question of whether whole organisms can be subject to de-extinction. Synthesis of whole genomes is fast becoming a reality, but the lack of close relatives able to bear young means that for this to be generally applicable, artificial cells and artificial wombs may be necessary and this seems to be a more distant scientific possibility as well as an ethically questionable undertaking [111]. Nevertheless, a project dubbed “Woolly Mammoth Revival” is already underway and for the time being, instead of synthesizing a complete mammoth genome, the genes within fibroblast cell cultures of the closely related Asian elephant species are being edited using the CRISPR-Cas9 technology [112] to introduce mutations believed to yield selected mammoth phenotypes such as long hair, large ears, altered haemoglobin and subcutaneous fat [113]. With the advent of direct cell-reprogramming techniques [114], trans-differentiation of fibroblasts into embryonic cells of such genetically engineered hybrids may be feasible though this is likely still a distant prospect even if challenges are satisfactorily addressed.

However, research at the molecular level is a more realistic possibility and indeed resurrection of long-lost proteins featuring differences to extant transcripts, which cater for the chemical characteristics of archaic habitats has been achieved. In some cases, this could have utility in the present; enzymes able to catalyse reactions in ancient earth conditions different from our own could conceivably have industrial or medical utility. For example, understanding how plants and animals in the past dealt with different oxygen and carbon dioxide levels may help us to discover new solutions to challenges arising from climate change. As technology advances further, it is likely that we will be able to recover ever more ancient molecules and genome sequence data, which may allow such insights.
